# Mitochondrial energetics is impaired in very long-chain acyl-CoA dehydrogenase deficiency and can be rescued by treatment with mitochondria-targeted electron scavengers

**DOI:** 10.1093/hmg/ddy403

**Published:** 2018-11-16

**Authors:** Bianca Seminotti, Guilhian Leipnitz, Anuradha Karunanidhi, Catherine Kochersperger, Vera Y Roginskaya, Shrabani Basu, Yudong Wang, Peter Wipf, Bennett Van Houten, Al-Walid Mohsen, Jerry Vockley

**Affiliations:** 1 Division Medical Genetics, Department of Pediatrics, University of Pittsburgh, Pittsburgh, PA, USA; 2 Programa de Pós-Graduação em Ciências Biológicas: Bioquímica, Departamento de Bioquímica, Instituto de Ciências Básicas da Saúde, Universidade Federal do Rio Grande do Sul, Porto Alegre, RS, Brazil; 3 Department of Pharmacology and Chemical Biology, University of Pittsburgh, Pittsburgh, PA, USA; 4 Department of Chemistry, University of Pittsburgh, Pittsburgh, PA, USA; 5 Department of Human Genetics, Graduate School of Public Health, University of Pittsburgh, Pittsburgh, PA, USA

## Abstract

Very long-chain acyl-CoA dehydrogenase (VLCAD) deficiency is the most common defect of mitochondrial long-chain fatty acid β-oxidation. Patients present with heterogeneous clinical phenotypes affecting heart, liver and skeletal muscle predominantly. The full pathophysiology of the disease is unclear and patient response to current therapeutic regimens is incomplete. To identify additional cellular alterations and explore more effective therapies, mitochondrial bioenergetics and redox homeostasis were assessed in VLCAD-deficient fibroblasts, and several protective compounds were evaluated. The results revealed cellular and tissue changes, including decreased respiratory chain (RC) function, increased reactive oxygen species (ROS) production and altered mitochondrial function and signaling pathways in a variety of VLCAD-deficient fibroblasts. The mitochondrially enriched electron and free radical scavengers JP4-039 and XJB-5-131 improved RC function and decreased ROS production significantly, suggesting that they are viable candidate compounds to further develop to treat VLCAD-deficient patients.

## Introduction

Very long-chain acyl-CoA dehydrogenase (VLCAD, EC: 1.3.99.3) controls the first transformation in the fatty acid oxidation (FAO) pathway and is a key enzyme for the energy metabolism in mitochondria. Individuals deficient in this enzyme (OMIM #609575) can present with a variety of clinical symptoms and a spectrum of severity that ranges from acute life-threatening illness in the newborn period to relatively mild disease first developing late in childhood or early adulthood. Two major phenotypes of VLCAD deficiency (VLCADD) in childhood have been recognized (1). The first consists of severe neonatal or early onset disease with recurrent episodes of hypoglycemia, acidosis, hepatic dysfunction and cardiomyopathy. Patients who survive their initial presentation can exhibit progressive cardiomyopathy, and have a reported 75% mortality rate in the first few years of life (2). In the second phenotype, children have later onset symptoms and can have repeated episodes of hypoketotic hypoglycemia, but are at low risk of developing cardiomyopathy, with a resultant lower mortality and better long-term prognosis. Regardless of the initial phenotype, recurrent rhabdomyolysis becomes a dominant feature in older children and adults. Multiple mutations have been identified in patients with VLCADD and some correlation of genotype with phenotype has been suggested (3). Patients with null mutations, leading to complete absence of VLCAD, tend to have more severe symptoms than those with some residual enzymatic activity (4).

The cellular pathophysiology responsible for causing the symptoms observed in patients with VLCADD has not been completely determined, but energy deficiency seems to play an important role, especially in the development of hypoglycemia and cardiomyopathy. In this regard, studies performed in animal models and patient cells indicate impairment of cellular energy metabolism and redox homeostasis (5,6). Other findings implicate an augmented inflammatory process related to rhabdomyolysis in VLCADD patients (7).

Treatment of patients consists mainly of restriction of dietary long-chain fats, and frequent meals to prevent catabolism (1,4). The replacement of long-chain natural fats by medium-chain triglycerides is also helpful since their metabolism bypasses the enzymes of long β-oxidation pathway (8–10). However, most patients continue to experience exercise intolerance and myalgia, with risk of episodic rhabdomyolysis (1,4). Carnitine is sometimes prescribed, but its use is controversial (11,12). Triheptanoin, a seven-carbon chain triglyceride shown to replenish tricarboxylic acid cycle (TCA) cycle intermediates in patients with VLCADD, is currently under clinical investigation (8,9). While this compound is effective in addressing hypoglycemia in patients, it is less so in treating or preventing cardiomyopathy, and only has a minor effect on recurrent rhabdomyolysis (8,13). These findings suggest that alternative cellular mechanisms may be relevant in the development of the latter two symptoms.

A new class of mitochondria-targeted electron and reactive oxygen species (ROS) scavengers has been recently described (14). These molecules consist of a nitroxide portion, with electron-, radical- and ROS-scavenging activities, and a targeting moiety that promotes their selective accumulation within mitochondria. Among these molecules, the two analogs JP4-039 and XJB-5-131 are based on the natural product gramicidin S and covalently linked to the antioxidant 4-amino-tempol (15–17). The mitochondrial-targeting sequence is reduced in JP4-039 compared with other GS-nitroxides, such as XJB-5-131, resulting in a lower degree of mitochondrial enrichment. Recent publications have shown that JP4-039 and XJB-5-131 are able to scavenge ROS and electrons escaping from the respiratory chain (RC), mitigate radiation damage, and prevent lipid peroxidation, apoptosis and ferroptosis (18–21). However, these compounds have not been evaluated as a potential therapy for inborn errors of energy metabolism.

The present study investigated potential additional mechanisms involved in the pathophysiology of VLCADD, including mitochondrial function and oxidative stress in fibroblasts of patients diagnosed with this disorder. Additionally, the effects of JP4-039 and XJB-5-131 on these parameters were examined as a test of the influence of redox homeostasis and as potential new treatment strategies for this disorder.

**Figure 1:**
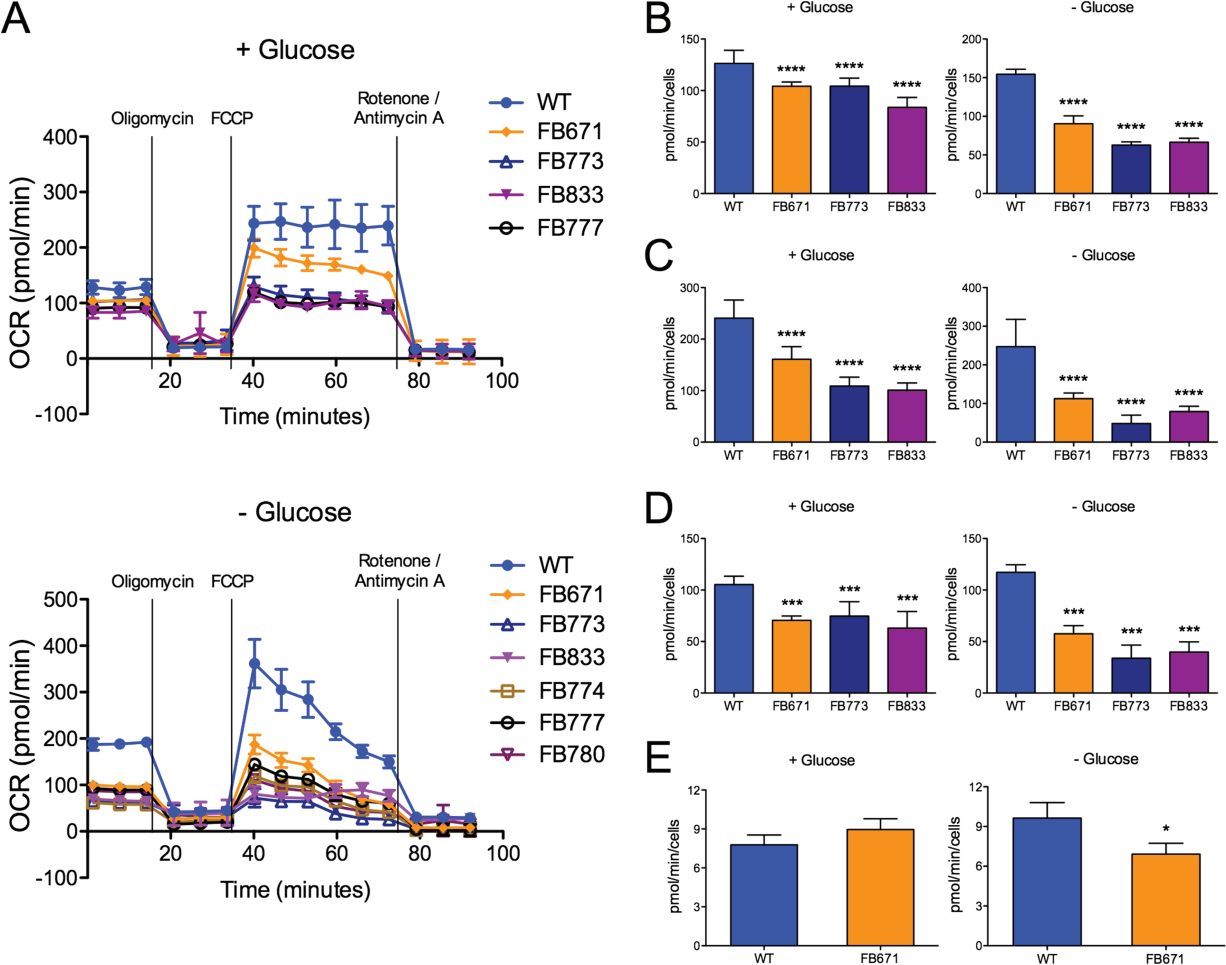
Mitochondrial respiration rates are lower in fibroblasts from VLCAD patients. Representative OCR of FB671, FB773, FB833, FB774, FB777 and FB780 fibroblasts cultured in media without glucose for 72 h prior to Seahorse analysis **(A)**. Basal respiration **(B)**, reserve capacity **(C)**, steady-state levels of ATP production **(D)** and oxygen consumption linked to ATP production **(E)** of FB671 fibroblasts cultured in media with or without glucose for 72 h (A, B, C and D) or 48 h **(E)**. Data are means ± SD. ^*^P < 0.05, ^***^P < 0.001, ^****^P < 0.0001, compared with WT (*t*-test for unpaired samples).

## Results

### Oxygen consumption and ATP production

**Figure 2:**
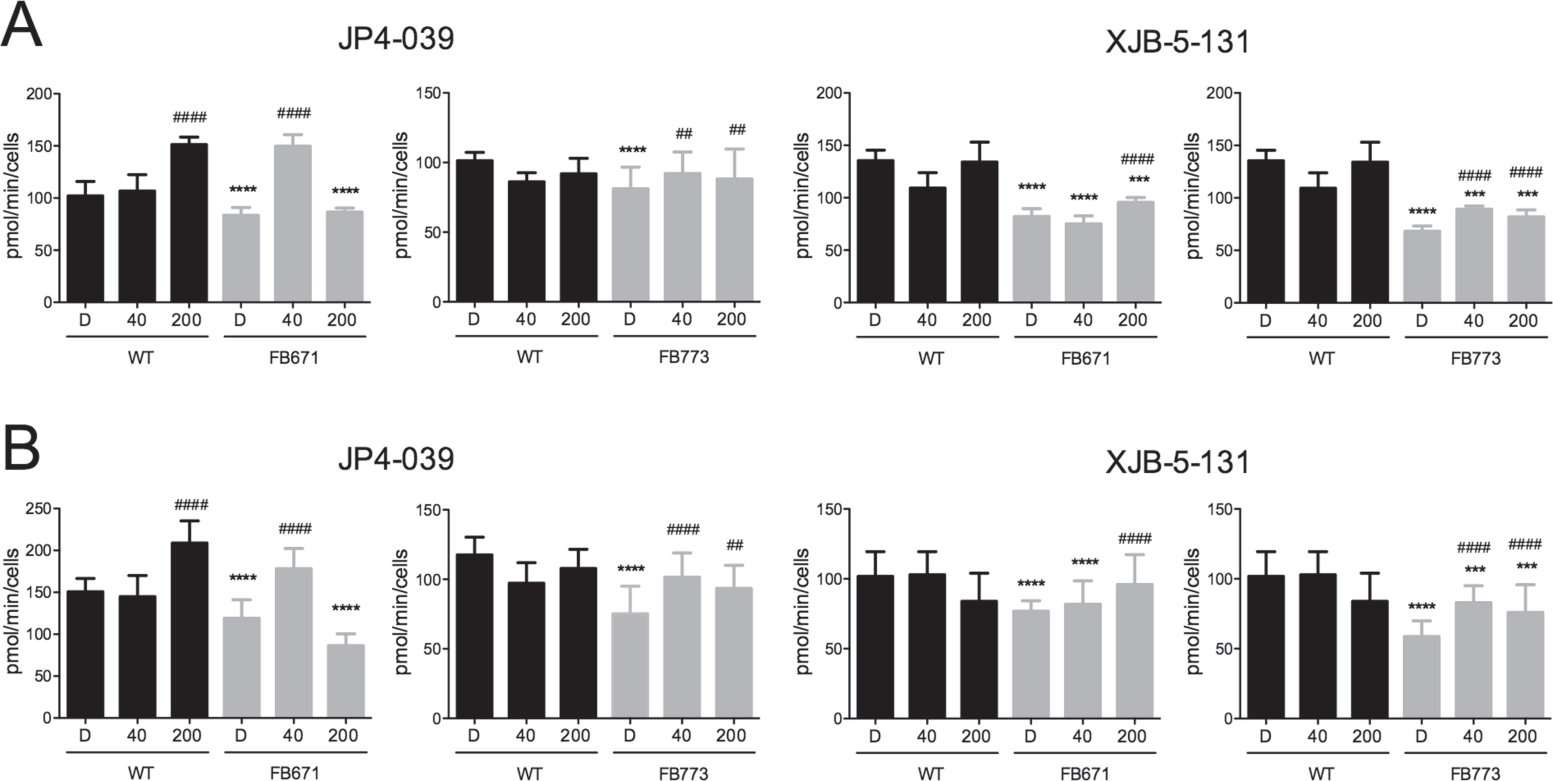
Effect of JP4-039 and XJB-5-131 on basal respiration **(A)** and reserve capacity **(B)** of FB671 and FB773 fibroblasts cultured in media without glucose for 72 h. Cells were exposed to DMSO (D), JP4-039 or XJB-5-131 (40 nM or 200 nM) for 24 h. Data are means ± SD. ^***^P < 0.001, ^****^P < 0.0001, compared with WT; ^##^*P* < 0.01, ^####^*P* < 0.0001, compared with FB671 or FB773 cells (Tukey multiple range test).

**Figure 3:**
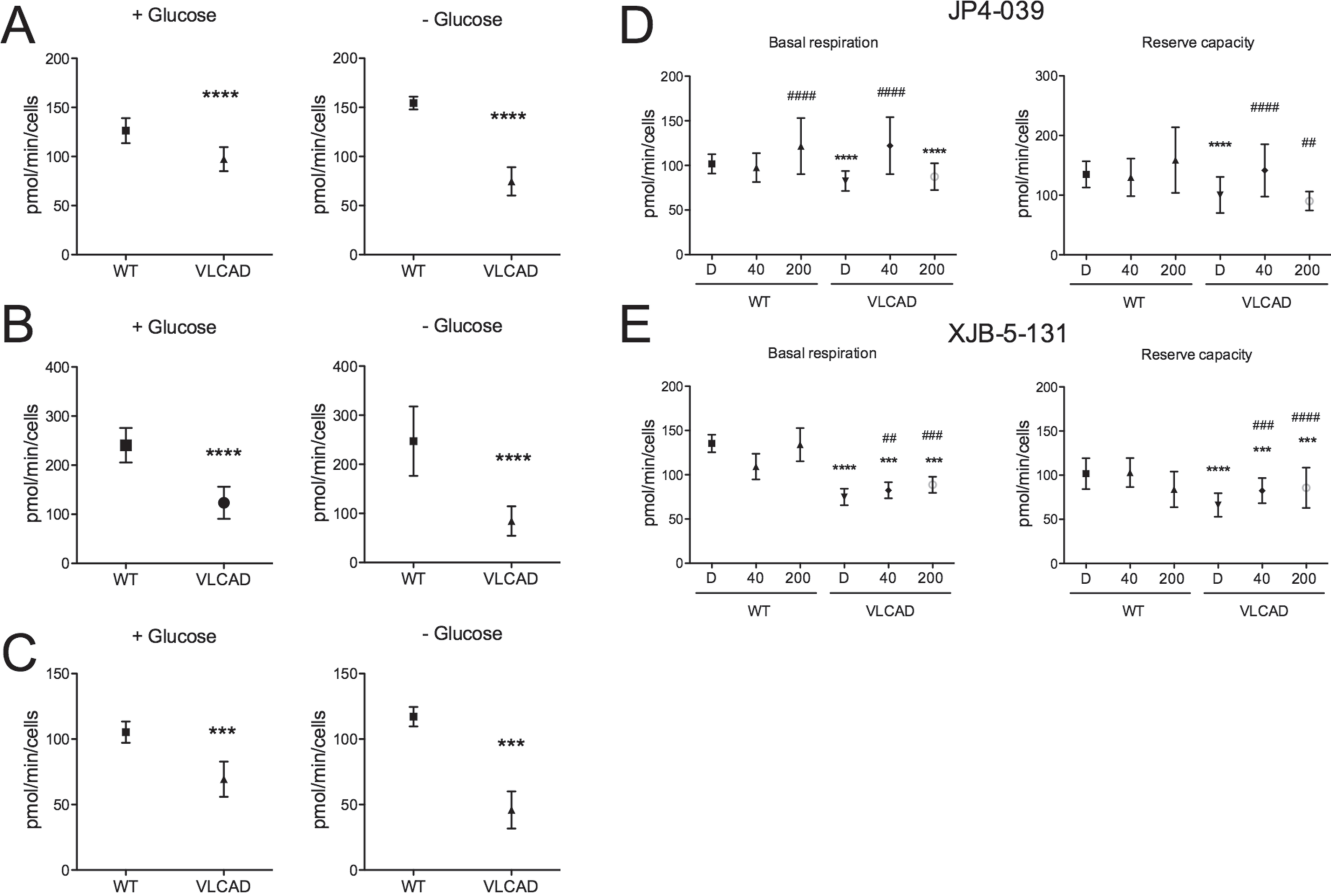
Representative OCR averaging three different VLCAD-deficient fibroblasts cultured in media with and without glucose for 72 h prior to Seahorse analysis. Basal respiration **(A)**, reserve capacity **(B)**, oxygen consumption linked to ATP production **(C)** and the effect of JP4-039 **(D)** and XJB-5-131 **(E)** on basal respiration and reserve capacity. Cells were exposed to DMSO (D), JP4-039 or XJB-5-131 (40 nM or 200 nM) for 24 h. Data are means ± SD. ^***^P < 0.001, ^****^P < 0.0001, compared with WT; ^##^*P* < 0.01, ^###^*P* < 0.001, ^####^*P* < 0.0001 compared with VLCAD cells (*t*-test for unpaired samples).

The bioenergetic state of the patient fibroblasts (FB671, FB773, FB833, FB777, FB774 and FB780) was measured by monitoring oxygen consumption in a Seahorse flux analyzer. Since cells grown in medium that contains high glucose can generate ATP through glycolysis that may mask deficiencies in oxidative phosphorylation (OXPHOS) in VLCAD patient samples, we used glucose-free medium for 72 h prior to these experiments. Basal respiration and reserve capacity were decreased in all VLCAD-deficient fibroblasts in normal media and without glucose (Fig. 1A). We have previously evaluated multiple control fibroblasts and settled on this sample as a consistent standard (22). A decrease of these parameters was also detected in FB671, FB773 and FB833 in normal media (Fig. 1A, B, C and D). ATP production was measured in FB671 cells to assess the consequences of reduced oxygen consumption. As shown in Figure 1E, a marked reduction in steady-state levels ATP levels was seen when cells were grown in the absence of glucose. Oxygen consumption linked to ATP production was also markedly decreased (Fig. 1D). Taken together, these data show energy homeostasis impairment in VLCAD-deficient fibroblasts. Since we observed altered mitochondrial function in VLCAD-deficient cells, we next assessed whether mitochondrial-targeted electron-scavenging compounds could ameliorate this mitochondrial dysfunction. To this end, FB671 and FB773 fibroblasts were treated with JP4-039 or XJB-5-131 for 24 h before evaluating basal respiration and reserve capacity. Both mitochondrial targeted antioxidants significantly increased basal respiration (Fig. 2A) and reserve capacity (Fig. 2B) in the two cultured fibroblasts. When we compared the averages of VLCAD-deficient cells with controls, we observed significant decrease in respiratory parameters (Fig. 3A, B and C), and an improvement of basal respiration and reserve capacity with JP4-039 and XJB-5-131 treatments (Fig. 3D and E, respectively).

### Mitochondrial mass, dynamics, membrane potential (ΔΨ) and citrate synthase activity

**Figure 4:**
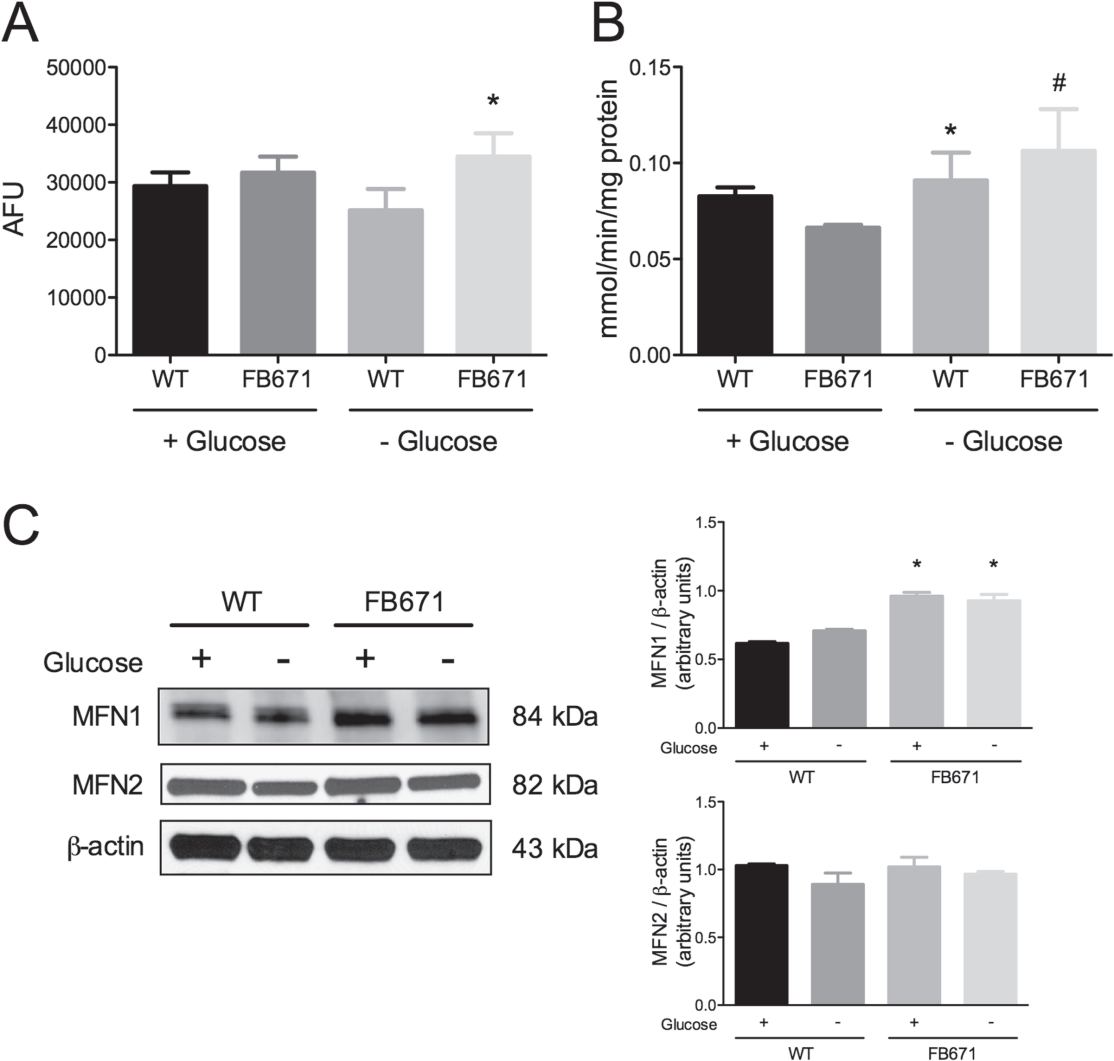
Mitochondrial mass **(A)**, CS activity **(B)** and mitofusins 1 and 2 (MFN1 and MFN2) protein content **(C)** in FB671 fibroblasts cultured in media with or without glucose for 48 h. For mitochondrial mass, VLCAD-deficient fibroblasts were incubated with MitoTracker Green. Data are means ± SD. ^*^P < 0.05, compared with WT; ^#^*P* < 0.05, compared with FB671 cells (*t*-test for unpaired samples).

Mitochondrial mass was evaluated in FB671 fibroblasts using the probe MitoTracker green. Figure 4A shows an increase in mitochondrial mass as compared to control human diploid fibroblasts (wild type; WT) cells when grown without glucose. Consistent with these results, the activity of citrate synthase (CS), the regulatory enzyme of the citric acid cycle, was increased in FB671 cells cultured in the absence of glucose (Fig. 4B). Since alterations in mitochondrial mass might occur due to changes in mitochondrial dynamics, we also measured the levels of MFN1, one of the main proteins involved in mitochondrial fusion. MFN1 content was increased in FB671 cells regardless of the presence of glucose (Fig. 4C). Next, we used the probe MitoTracker Red to assess the ΔΨ in FB671 fibroblasts. No significant alterations on ΔΨ were verified with or without glucose in the growth media (Supplementary Material, Fig. S1).

### FAO flux, VLCAD content and activity

**Figure 5:**
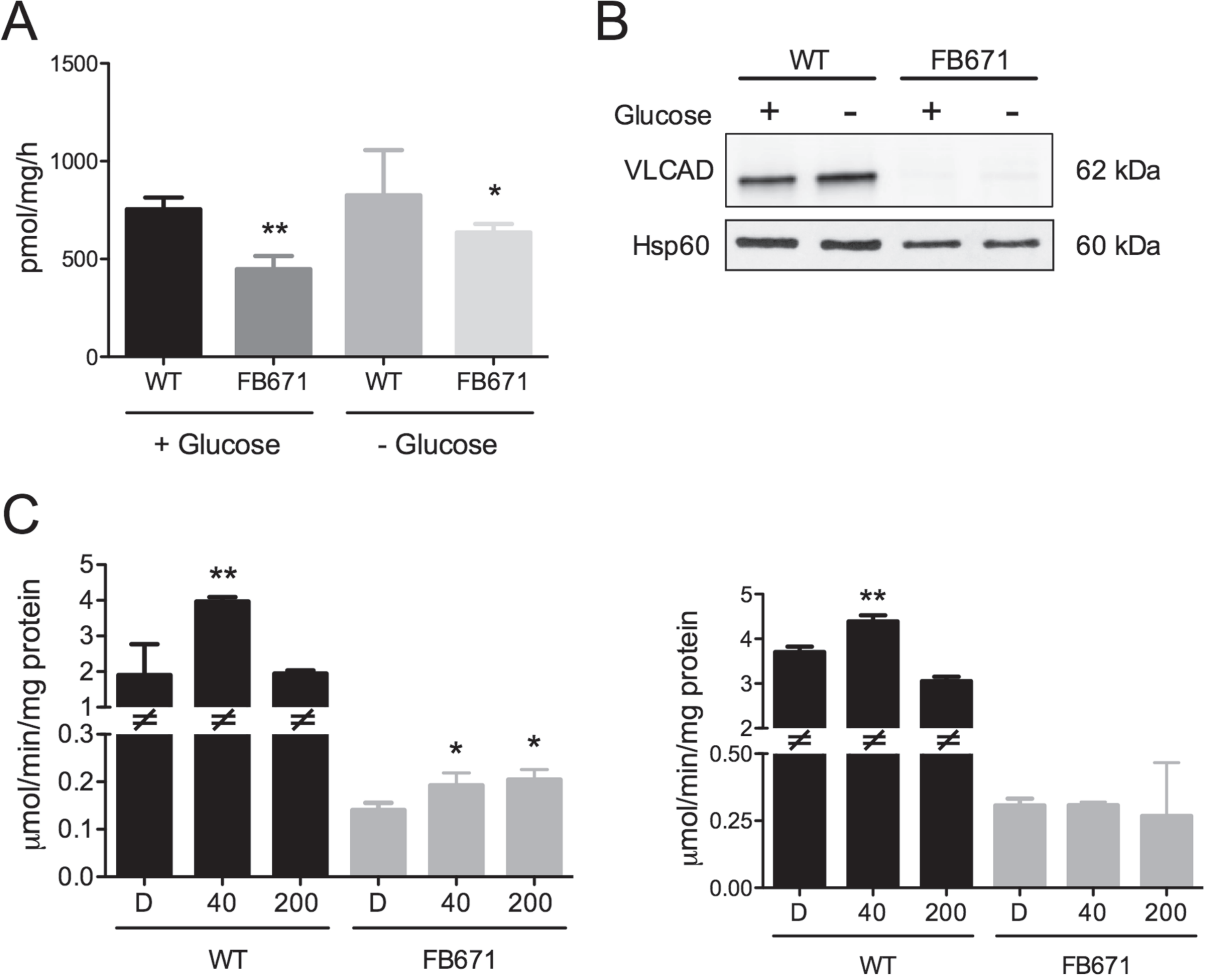
FAO flux **(A)** and VLCAD protein content **(B)** and activity **(C)** in FB671 fibroblasts cultured in media with or without glucose for 48 h. FAO flux was measured in fibroblasts cultured in a 6-well plate (A). VLCAD content was measured in mitochondria prepared from fibroblasts. The image was electronically adjusted to optimize comparisons within a single gel but not for comparisons across different gels. High contrast and overexposed images were not utilized (B). VLCAD activity was measured in whole cell lysates. Cells were exposed to JP4-039 or XJB-5-131 (40 nM or 200 nM) during 24 h (C). Data are means ± SD. ^*^P < 0.05, ^**^P < 0.01 compared with WT or untreated (*t*-test for unpaired samples).

VLCADD has been shown to severely reduce flux through the FAO pathway. We sought to confirm this deficit in patient cells and evaluate the effect of treatment on flux. Flux through the FAO pathway in FB671 fibroblasts was decreased in the presence or absence of glucose as demonstrated by reduced oxidation of palmitate (Fig. 5A). In line with this finding, VLCAD protein (Fig. 5B) was markedly decreased in FB671 cells as was its activity (Fig. 5C), regardless of the presence of glucose during growth. It is important to note that VLCAD enzyme activity was measured with its optimum substrate palmitate (C16-CoA) using the highly specific and sensitive electron transfer flavoprotein (ETF) fluorescence reduction assay (23). No other acyl-CoA dehydrogenases are present in fibroblasts that can utilize palmitate as substrate, and the very low residual activity in the absence of a visible VLCAD on western blotting is an indication of the high sensitivity of the enzyme assay. Treatment with JP4-039, but not with XJB-5-131, slightly increased VLCAD activity (Fig. 5C).

### ROS production

**Figure 6:**
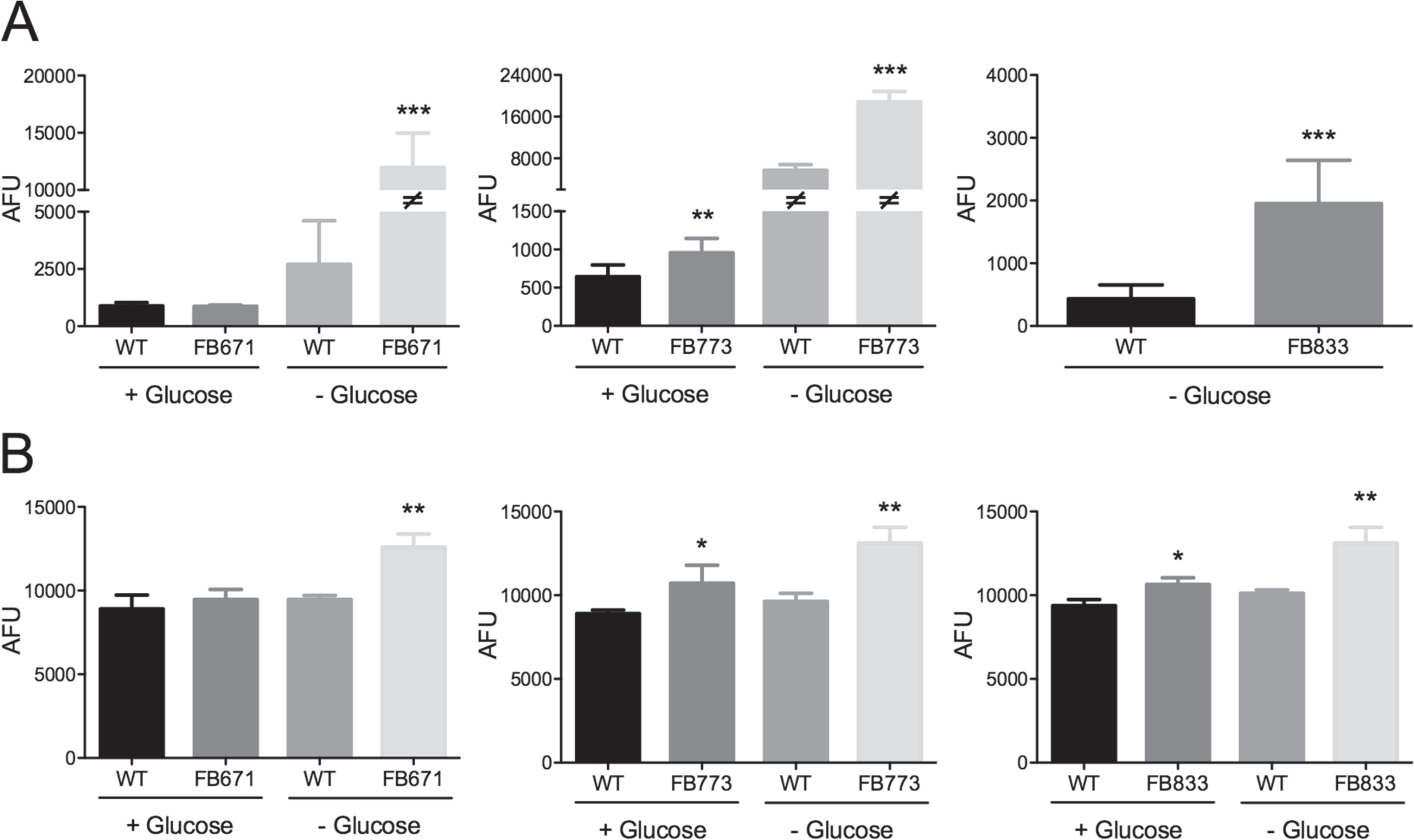
Superoxide production in FB671 **(A)**, FB773 **(B)** and FB833 **(C)** fibroblasts cultured in media with or without glucose for 48 h. ROS in FB671 **(D)** and FB833 **(E)** fibroblasts cultured in media with or without glucose for 48 h. VLCAD-deficient fibroblasts were incubated with MitoSOX Red (A, B and C) or the probe DCFH (D and E). Data are means ± SD. ^*^P < 0.05, ^**^P < 0.01, ^***^P < 0.001, compared to WT (*t*-test for unpaired samples).

**Figure 7:**
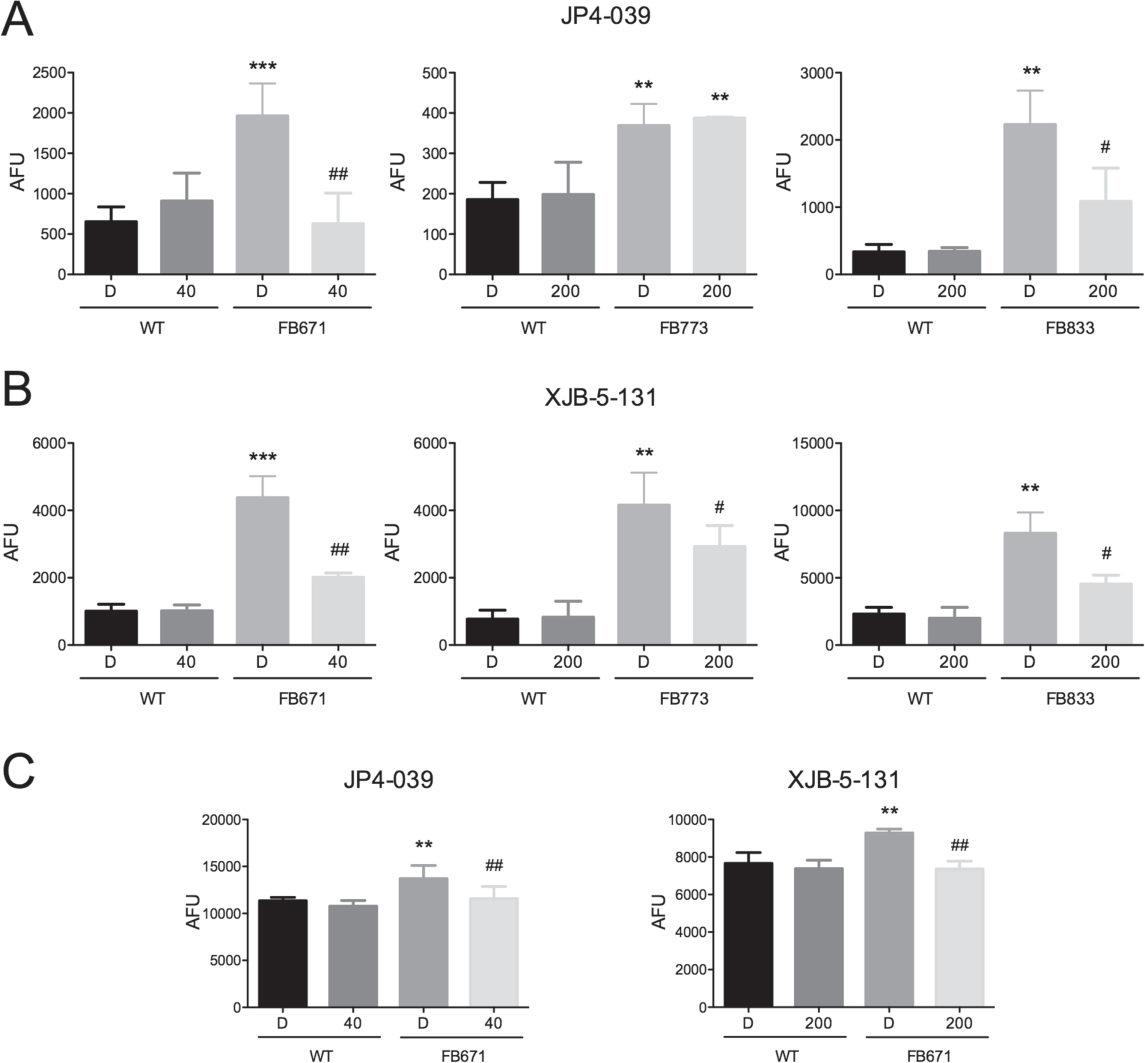
Effect of JP4-039 and XJB-5-131 on ROS levels in FB671, FB773 and FB833 fibroblasts cultured in media without glucose for 48 h. VLCAD-deficient cells were exposed to JP4-039 or XJB-5-131 (40 or 200 nM) during 24 h **(A–C)**. ROS levels were measured with probe MitoSOX Red (A and B) or the probe DCFH (C). Data are means ± SD. ^**^P < 0.01, ^***^P < 0.001, compared with WT; ^#^*P* < 0.05, ^##^*P* < 0.01, compared with FB671, FB773 or FB833 cells (*t*-test for unpaired samples).

Mitochondrial RC dysfunction has been shown to lead to increased ROS generation (22), but ROS production has not been examined in the context of FAO deficiency. Given the impaired oxygen consumption in VLCAD deficient-cells, we measured superoxide levels with MitoSOX Red in patient fibroblasts FB671, FB773 and FB833. A significant increase of superoxide levels was observed in all VLCAD-deficient cells when cultured in the absence of glucose (Fig. 6A). We next evaluated the effect of treatment of VLCAD-deficient cells with bezafibrate (BEZ), *N*-acetylcysteine (NAC), resveratrol (RESV), MitoQ, trolox, JP4-039 and XJB-5-131 on superoxide production. While JP4-039 and XJB-5-131 decreased superoxide anion radical generation in FB671, FB773 and FB833 (Fig. 7A and B), the other compounds did not change the levels of this free radical (Supplementary Material, Table S2). Increased cellular ROS was confirmed in FB671, FB773 and FB833 cell extract with the probe dichlorofluorescein (DCFH) (Fig. 6B), which is sensitive to all ROS, not just superoxide (24), and JP4-039 and XJB-5-131 similarly decreased the DCFH signal (Fig. 7C).

**Figure 8:**
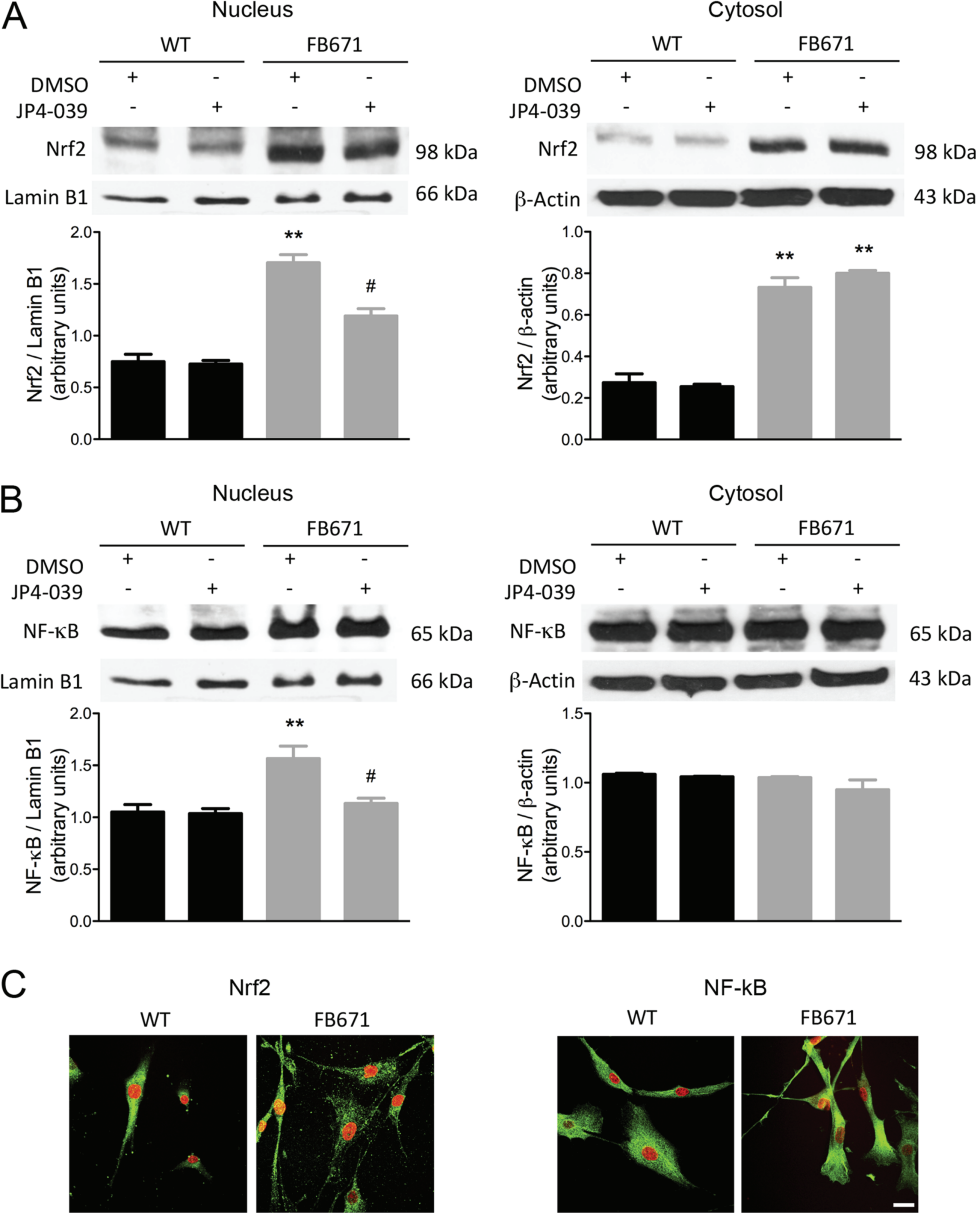
Nrf2 **(A)** and NF-κB **(B)** protein content in nucleus and cytosol prepared from FB671 fibroblasts, normalized by the content of the proteins lamin B1 (nucleus) or β-actin (cytosol). Fibroblasts were cultured in media without glucose for 48 h and treated with DMSO or JP4-039 (40 nM) for 24 h. Representative images show colocalization of Nrf2 or NF-κB protein visualized with green fluorescently tagged antibody and nuclei visualized with DAPI staining as yellow (white arrows). Calibration bar indicates 50 μm **(C)**. ^**^P < 0.01, compared with WT; ^#^*P* < 0.05, compared with FB671 (*t*-test for unpaired samples).

**Figure 9:**
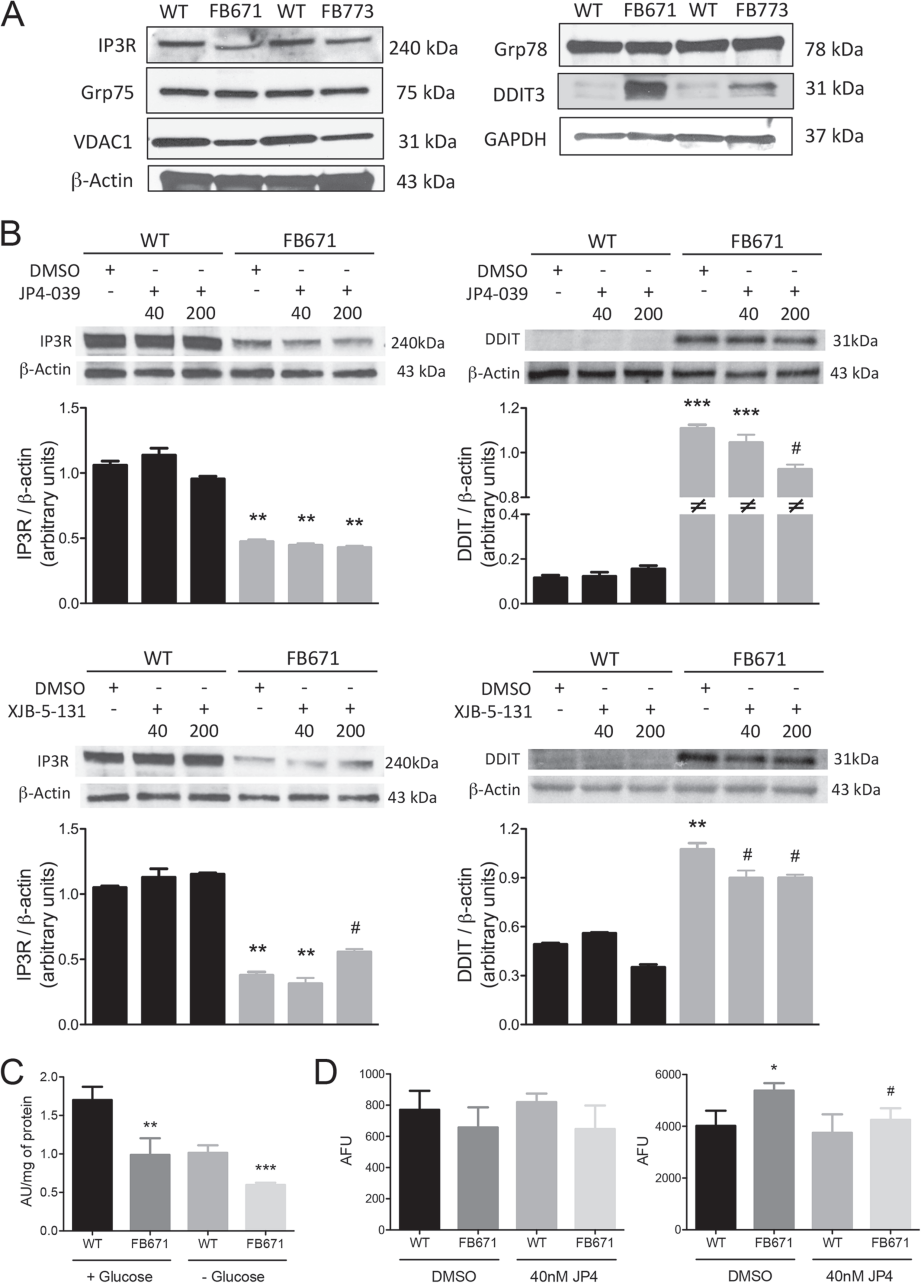
IP3R, Grp75, VDAC1, Grp78 and DDIT3 protein content in cell lysates prepared from VLCAD-deficient fibroblasts cultured in media without glucose **(A)**. VLCAD-deficient cells were exposed to JP4-039 or XJB-5-131 (40 or 200 nM) during 24 h before evaluating protein content **(B)**. Cell viability **(C)** and apoptosis **(D)** in FB671 fibroblasts cultured in media with or without glucose for 48 h. Cell viability were measured spectrophotometrically in fibroblasts cultured in a 96-well plate (B). Apoptosis was measured by flow cytometry after incubation with Annexin V and PI. VLCAD-deficient cells were exposed to JP4-039 (40 nM) during 24 h before evaluating apoptosis (D). Data are means ± SD. ^*^P < 0.05, ^**^P < 0.01, ^***^P < 0.001, compared with WT; ^#^*P* < 0.05, compared with untreated FB671 cells (*t*-test for unpaired samples).

### Protein expression of the transcription factors Nrf2 and NF-κB

In light of the evidence of oxidative stress in the VLCAD-deficient patient fibroblasts, we evaluated the level of nuclear factor (erythroid-derived 2)-like 2 (Nrf2) and nuclear factor kappa-light-chain-enhancer of activated B cells (NF-κB)—two important transcription factors involved in redox homeostasis and control of inflammation. Increased nuclear Nrf2 (Fig. 8A) and NF-κB (Fig. 8B) protein levels were observed in FB671 fibroblasts as compared with WT fibroblasts. The cytosolic content of Nrf2 was also increased in FB671 cells (Fig. 8A), while the NF-κB cytosolic levels were not altered (Fig. 8B). JP4-039 treatment of cells led to a slight intensity decrease of these transcription factors (Fig. 8).

### Protein content of ER-mitochondria crosstalk

Alterations in endoplasmic reticulum (ER)-mitochondria crosstalk and ER stress are closely related to impairment of mitochondrial function (25). Thus, we measured the quantity of proteins involved in this crosstalk [inositol 1,4,5-trisphosphate receptor 3 (IP3R), voltage-dependent anion channel 1 (VDAC1) and glucose-related protein 75 (Grp75)], as well as ER stress [DNA damage inducible transcript 3 (DDIT3) and Grp 78 (Grp78)], in VLCAD-deficient fibroblasts FB671 and FB773 grown without glucose. VDAC1 and IP3R were decreased, whereas DDIT3 was increased in both cultured fibroblasts as compared with WT fibroblasts (Fig. 9A). In contrast, Grp75 and Grp78 were not altered in either cell line (Fig. 9A). JP4-039 or XJB-5-131 treatment did not modify IP3R expression in FB671 fibroblasts, but slightly decreased DDIT3 content (Fig. 9B).

### Cell viability and apoptosis

The ultimate cause of muscular symptoms in patients with VLCADD is unknown. Treatment with triheptanoin is hypothesized to replete the TCA cycle and restore more normal bioenergetics in long-chain FAO defects. However, patients still experience muscular symptoms (rhabdomyolysis) and to a lesser extent, cardiomyopathy. Given the reduction of (OXPHOS) and production of ROS, we hypothesized that cells with VLCADD would be prone to apoptosis, regardless of the availability of glucose for glycolysis. To study this possibility, we evaluated cell viability in FB671 fibroblasts with a 3-(4,5-dimethylthiazol-2-yl)-5-(3-carboxymethoxyphenyl)-2-(4-sulfophenyl)-2H-tetrazolium (MTS) reduction assay. Decreased cell viability was seen in these fibroblasts regardless of the presence of glucose in the growth media (Fig. 9C). Apoptosis measured with an annexin V assay was increased in FB671 fibroblasts in the absence of glucose, as compared with WT cells, and treatment with JP4-039 significantly decreased cell death (Fig. 9D).

## Discussion

In this study, we demonstrated direct impairment of global mitochondrial bioenergetics and function in fibroblasts from patients with VLCADD. Regardless of mutation, VLCAD-deficient cells showed a marked decrease of mitochondrial RC function and ATP levels that tended to be exaggerated by growth in glucose-free media. Although the *ACADVL* gene mutations were heterogeneous in the examined fibroblasts, impairment in oxygen consumption was observed in all cultured cells, which is in line with observations showing that long-chain fatty acids accumulated in VLCADD impairs bioenergetics in mitochondria of rat brain (26). These findings are accompanied by increased ROS production measured with an *in vivo* probe and an *in vitro* technique. Indirect evidence has previously suggested that VLCAD-deficient cells have increased ROS production, and other indirect evidence of energy dysfunction associated with FAO defects has been published (6,27–31). FAO is a major energy-producing pathway during times of stress that provides electrons directly to the electron transport chain (ETC) for ATP production. In addition, FAO is particularly critical for cardiac tissue, which uses fatty acids for ~80% of its energy needs even in the fed state. Thus, secondary impairment of (OXPHOS) will exacerbate the primary energy deficit of VLCADD. It is well established that mitochondria generate superoxide and/or hydrogen peroxide from various sites associated with substrate catabolism and (OXPHOS), and that these different sites have various roles in signaling and disease (32,33). The main sources of cellular superoxide are the mitochondrial ETC complexes I and III, from which small leaks of electrons occur during OXPHOS, but leaks from ETF/ETF-QO have also been implicated (34,35). High levels of superoxide lead to molecular damage that exceeds cellular repair capacity, triggering cellular dysfunction (32). The potential clinical implications of a more complex energy defect in cells and tissue in the face of VLCADD are considerable, as they suggest a pathophysiology beyond the scope of the primary FAO defect. We have previously reported that the proteins involved in FAO and ETC are physically associated, suggesting that the induced ETC deficiency in VLCADD could result from disruption of an energy complex as well as simple reduction of ETC substrates (32,36). Moreover, previous works have shown cellular toxicity induced by the major accumulated long-chain fatty acids and acylcarnitines in VLCADD. At high concentrations, these metabolites provoke mitochondrial dysfunction by different mechanisms, including calcium homeostasis disturbance, mitochondrial membrane potential dissipation, OXPHOS uncoupling and apoptosis (26,37).

Since mitochondria are dynamic organelles whose morphology is maintained at least in part by the equilibrium between fusion and fission, we investigated the protein content of MFN1, the main protein involved in the fusion process and mitochondrial mass. VLCAD-deficient cells had a significant increase in MFN-1 levels and mitochondrial mass compared to WT fibroblasts. These changes may represent an adaptation to decreased energy production (38). This is in accordance with data showing that intracellular fatty acid accumulation as occurs in FAO deficiency results in cell toxicity and promotes activation of the PPARα promotor, thus inducing mitochondrial biogenesis (39). Corroborating our data, a recent publication also showed mitochondrial morphology and connectivity changes in fibroblasts from VLCAD patients (40). Mitofusins can also transiently increase the permeability of the outer mitochondrial membrane, and facilitate formation of the mitochondrial transition pore if it occurs concomitantly with loss of permeability at the inner mitochondrial membrane under stress conditions (41,42).

The increase in the activity of CS reinforces the hypothesis that mitochondrial biogenesis is induced in VLCAD-deficient cells. An increase in this Krebs cycle enzyme that provides additional reducing equivalents for the ETC may also be a compensatory mechanism for the bioenergetics failure in the FAO pathway and the ETC as suggested by the reduced oxygen consumption in VLCAD-deficient cells (43,44).

Activation of Nrf2 and NF-κB signaling pathways may also be triggered in VLCAD-deficient cells by alterations in the redox state related to ETC dysfunction. Nrf2 is a transcription factor that behaves as a primary sensor of oxidative stress and as a regulator of the antioxidant system due to its ability to modulate the expression of numerous antioxidant and detoxifying genes (45). We observed increased Nrf2 expression in both cellular compartments, cytosol and nucleus, which indicates that this transcription factor is being translocated to the nucleus. This is in accordance with our results showing increased ROS production, which is known to oxidize Keap1, an Nrf2 inhibitory protein, and therefore induce nuclear translocation of this transcription factor (46). Increased expression of NF-κB was also seen in VLCAD-deficient cells, mainly in the nucleus; NF-κB belongs to a family of transcription factors that mediates several different immune responses. The NF-κB signaling pathway is activated with intracellular redox state disturbances and inflammatory processes (47,48), implying inflammation induction in VLCAD-deficient cells.

**Figure 10:**
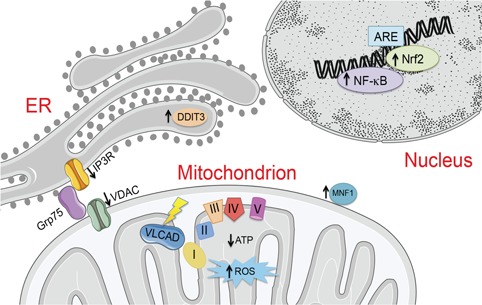
Seminotti *et al.* describe mitochondrial and redox homeostasis dysfunction in VLCAD-deficient fibroblasts, as well as impairment of endoplasmic reticulum-mitochondria crosstalk, induced by the primary FAO defect. Treatment with the mitochondrially enriched free radical and electron scavengers JP4-039 and XJB-5-131 improved RC function and decreased ROS production.

Mitochondrial bioenergetic disturbance has recently been shown to disrupt communication between the rough ER and mitochondria in cells, inducing ER stress (25,49,50). This crosstalk plays an important role in calcium homeostasis, mediating calcium translocation from ER to mitochondria through the interaction of mitochondrial VDAC with IP3R on the ER via the chaperone Grp75 (Fig. 10) (51,52). In our VLCAD-deficient cells, we observed decreased levels of VDAC1 and IP3R, suggesting a disruption of ER-mitochondria crosstalk. ER stress, reflected by increased levels of DDIT3, was also seen in VLCAD-deficient cells. Upregulation of DDIT3 is consistent with the apoptosis increase observed in these cultured fibroblasts since it is a transcription factor that modulates this process. Therefore, we speculate that mitochondria in VLCAD-deficient cells undergo irreversible morphologic changes that lead to apoptosis (53).

Accumulation of ROS in mitochondria is a common pathogenic finding in many disorders of the ETC but has not been broadly recognized in FAO disorders. Unfortunately, treatment with traditional antioxidants has not been effective in ETC deficiencies, at least in part due to their poor penetration into mitochondria and mitochondrial membranes. In contrast, JP4-039 and XJB-5-131 have structural motifs that target them with much higher efficiency to mitochondria, and have been shown to ameliorate cellular damage induced by mitochondrial ROS accumulation diseases *in vitro* and *in vivo* (19,54–57). Treatment with JP4-039 and XJB-5-131 considerably improved the oxygen consumption rate (OCR) and reduced ROS in VLCAD-deficient fibroblasts, but not to the same extent in all cases. While JP4-039 shows significantly lower enrichment into mitochondria compared with XJB-5-131 (21), the variability in response among the different fibroblasts may also reflect other factors, such as the extent of structural perturbations in the fatty acid β-oxidation complex induced by the various mutations, as well as the pharmacodynamics of these compounds and the rate of their interactions with ROS. Furthermore, expression of Nrf2 and NF-κB was decreased after JP4-039 treatment, presumably due to neutralization of pro-inflammatory and pro-apoptotic cellular responses. More importantly, JP4-039 partially increased VLCAD activity. To our knowledge, this is the first demonstration of an improvement in important cellular functions that reflect energy homeostasis by treatment with an antioxidant, and highlights the potential use of these compounds as a therapy for VLCADD, as well as potentially other long-chain FAO disorders.

The nitroxide moieties in JP4-039 and XJB-5-131 are effective in catalyzing the dismutation of superoxide anions and other reactive species generated in mitochondria, including those formed by electron leakage from OXPHOS (58,59). Our data suggest that the excess production of ROS in VLCAD enzyme could establish a negative feedback loop by damaging the ETC and further exacerbating ROS production. In this context, a previous report demonstrated that decreasing mitochondrial detectable reactive species prevents mitochondrial DNA damage and ultimately improves ETC function (60). Moreover, in accordance with our results, prior studies demonstrated that targeted nitroxides, including JP4-039, work at the level of the mitochondria to inhibit caspase-3 expression and apoptosis (19,61,62).

In summary, we demonstrate a marked bioenergetic impairment in fibroblasts from patients with VLCADD with a significant increase in superoxide production. Our findings also implicate a redox status disturbance and inflammation in the cellular injury observed in this disorder. Due to the molecular heterogeneity VLCADD (2), multiple strategies for therapy are likely to be helpful and may need to be guided by genotype.

## Materials and Methods

Experiments were performed in accordance with the approved guidelines and regulations. Experimental human protocols were approved by the Institutional Review Board at the University of Pittsburgh, protocol #404017.

### Subjects

Cultured skin fibroblasts (FB671, FB773, FB833, FB777, FB774 and FB780) with different mutations in the *ACADVL* gene were obtained from patient skin biopsies, while control fibroblast cells (WT) were obtained from three anonymous healthy individuals (Supplementary Material, Table S1). Biopsies from patients were performed on a clinical basis with written informed consent from patients and/or parents.

### Cell culture and treatments

Cells were routinely grown in Dulbecco’s Modified Eagle Medium (DMEM; Corning Life Sciences, Manassas, VA), containing 10% glucose, or in DMEM devoid of glucose for 48–72 h. Both media were supplemented with 10% fetal bovine serum, 4 mM glutamine, 100 IU penicillin and 100 μg/mL streptomycin (Corning Life Sciences). The passage number of the fibroblasts used in the experiments was 3–5 for WT cells and 4–7 for VLCAD-deficient cells.

After measuring the respiratory parameters in six different VLCAD-deficient fibroblasts, we decided to perform further experiments with FB671. We also examined ROS levels in FB773 and FB833 for measuring.

Cells were treated with experimental compounds at various concentrations 24 or 48 h before the assays. The compounds used were NAC (1 mM) (Sigma-Aldrich, St Louis, MO), BEZ (600 μM) (Sigma-Aldrich), RESV (75 μM) (Sigma-Aldrich), MitoQ (200 nM) (MitoQ Ltd, Auckland, New Zealand), Trolox (a hydrosoluble analogue of vitamin E; 1 mM) (Sigma-Aldrich), JP4-039 (40 and 200 nM) and XJB-5-131 (40 and 200 nM), obtained from Dr Peter Wipf, Department of Chemistry, University of Pittsburgh (16,17). All compounds were prepared in dimethylsulfoxide (DMSO), except for NAC that was prepared in media.

### Measurement of mitochondrial respiration

OCR was measured with a Seahorse XF^e^96 Extracellular Flux Analyzer (Agilent, Santa Clara, CA). Cells were seeded in 96-well Seahorse tissue culture microplates in growth media at a density of 80 000 cells per well. To ensure equal cell numbers, cells were seeded in cell culture plates pre-coated with Cell-Tak (BD Biosciences, San Jose, CA). All cultured fibroblasts were measured with four to six wells per cell. Then, the entire experiment was repeated. Before the Seahorse assay, cells were incubated for 1 h without CO_2_ in unbuffered DMEM. Initial OCR was measured to establish a baseline at the resting state (basal respiration) followed by injection of oligomycin (an inhibitor of ATP synthase) that reduces OCR, representing ATP turnover. Subsequent injection of 300 nM carbonyl cyanide 4-(trifluoromethoxy)phenylhydrazone (FCCP, Seahorse XF Cell Mito Stress Test Kit; Agilent, Santa Clara, CA) dissipates the proton gradient and allows maximum respiration. The rise in OCR upon FCCP addition represents mitochondrial reserve capacity. Finally, rotenone and antimycin A were added to effectively disable the ETC and inhibiting the total mitochondrial respiration. The remaining OCR represents non-mitochondrial respiration. The difference between oligomycin- and rotenone and antimycin A-responsive OCR reflects proton leak (see Fig. 1A for more details). Data are reported in pmol of O_2_ reduced/min.

### ATP assay

Steady-state levels of ATP were measured with a bioluminescence assay kit (ATPlite™; PerkinElmer Inc., Waltham, MA), according to the manufacturer’s instructions. Luminescence was quantitated in a SpectraMax® i3x Platform multi-mode microplate reader system (Molecular Devices, LLC, Sunnyvale, CA). Data are reported in μmol of ATP/mg of protein.

### Mitochondrial membrane mass and superoxide production

Cell suspensions containing 1 × 10^5^ cells/mL were incubated for 25 min at 37°C with 150 nM (Mitotracker Green; Invitrogen, Grand Island, NY) for mitochondrial mass evaluation or for 15 min at 37°C with 5 μM (MitoSOX Red; Invitrogen) for superoxide production measurement (63). After incubation, samples of 10 000 cells were analyzed in a Becton Dickinson FACSAria II flow cytometer (BD Biosciences).

### ROS production

Cell suspensions containing 1 × 10^5^ cells/mL were incubated for 30 min at 37°C with 150 nM DCFH (Invitrogen) for reactive species production. After incubation, samples of 10 000 cells were analyzed in a Becton Dickinson FACSAria II flow cytometer (BD Biosciences).

### Western blot

Cells were grown in T175 flasks and, at 90–95% confluence, were harvested by trypsinization, then pelleted and stored at −80°C for western blot analysis. Protein content in samples was quantified for data normalization using *DC*™ Protein Assay kit (Bio-Rad Laboratories, Hercules, CA).

### Mitochondria preparation

Cell pellets were resuspended in 150–250 μL of 5 mM Tris buffer; pH 7.4, containing 250 mM sucrose; 2 mM EDTA, protease inhibitor cocktail (Roche Diagnostics, Mannheim, Germany); and 0.5 μM trichostatin A (Sigma-Aldrich), then homogenized and centrifuged at 1000 × *g* for 5 min at 4°C. The pellet was discarded and the supernatant centrifuged at 12 000 g for 15 min at 4°C. The pellet containing mitochondria was resuspended in 50 mM Tris buffer and pH 7.4, sonicated and centrifuged again at 14 000 × *g* for 15 min at 4°C. The supernatant was then used for western blotting as previously described (64). Briefly, 10–20 μg of protein was loaded onto the gel. Following electrophoresis, the gel was blotted onto a nitrocellulose membrane, which was incubated with mouse anti-mitofusin 1 (MFN1) monoclonal antibody (1:100) (Abcam, Cambridge, MA), mouse anti-mitofusin 2 (MFN2) monoclonal antibody (1:100) (Abcam), mouse anti-dynamin-related protein 1 (DRP1) monoclonal antibody (1:100) (Abcam), rabbit anti-VLCAD antiserum (1:1000) (Cocalico Biologicals Inc., PA), rabbit anti-VDAC1 monoclonal antibody (1:1000) (Abcam), mouse anti-Grp75 monoclonal antibody (1:250) (Abcam), rabbit anti-Grp78 polyclonal antibody (1:250) (Abcam), mouse anti-DDIT3 monoclonal antibody (1:250) (Abcam), goat anti-IP3R polyclonal antibody (1:50) (Santa Cruz Biotechnology, Dallas, TX) or IgG-HRP conjugated antibody (Bio-Rad Laboratories). Staining of the membranes with Ponceau S (Sigma-Aldrich) or mouse anti-β-actin monoclonal antibody (1:10 000) (Sigma-Aldrich), or mouse anti-glyceraldehyde 3-phosphate dehydrogenase monoclonal antibody (1:15 000) (Abcam), was used to verify equal loading.

### Nuclear and cytosolic fraction preparation

Cell pellets were washed with cold phosphate-buffered saline, lysed with a pre-cooled homogenizer in 300 μL cold buffer (10 mM HEPES, 1.5 mM MgCl_2_, 1 mM KCl and 1 mM DTT) plus 1 μg/μL protease, phosphatase inhibitor cocktail, 1 mM phenylmethanesulfonyl fluoride (PMSF) and 0.5% Nonidet P-40, and incubated on ice for 15 min. The homogenates were centrifuged at 850 × *g* for 10 min at 4°C and the supernatants (cytoplasmic extracts, SN1) were collected and stored at −80°C. The pellets were resuspended in 200 μL of cold buffer, transferred to pre-cooled microcentrifuge tubes and incubated on ice for 15 min. Then, 0.5% Nonidet P-40 was added and the samples were incubated on ice for 5 min and mixed for 10 s. The suspensions were centrifuged at 14 000 × *g* for 30 s at 4°C and the supernatants were collected in SN1, then, the pellets were resuspended in 50 μL of complete lysis buffer (20 mM HEPES, 1.5 mM MgCl_2_, 0.2 mM EDTA, 20% glycerol, 420 mM NaCl and 1 mM DTT), plus 1 μg/mL protease, phosphatase inhibitor cocktail and 1 mM PMSF, mixed for 10 s and incubated on ice for 40 min (mixed for 10 s each 5 min). Finally, the suspensions were mixed for 30 s and centrifuged at 14 000 × *g* for 10 min at 4°C. The supernatants (nuclear extracts, SN2) were collected and stored at −80°C. Inmunodetection was performed using the following primary antibodies, according to datasheet specifications: anti-Nrf2 antibody (1:500) (Abcam), anti-NF-κB p-65 antibody (1:500) (Abcam), anti-Lamin B1 (1:1000) (Abcam) and anti-β-actin (1:1000) (Santa Cruz Biotechnology).

### Immunofluorescence microscopy

Fibroblasts were seeded at a concentration of 1 × 10^5^ cells/mL on glass cover slips pre-treated with poly-D-lysine in a 12-well plate and allowed to grow overnight at 37°C in a 5% CO_2_/95% humidity incubator. After 80–90% confluence, cells were incubated with the antibodies anti-VLCAD (1:1000), anti-Nrf2 (1:100) or anti-NF-κB (1:1000) at 4°C overnight. After brief washing with TBST, cells were incubated with donkey anti-rabbit secondary antibody Alexa Fluor 488 (Invitrogen). Nuclei were immunostained with DAPI. The coverslips were then mounted before acquiring images with an Olympus Confocal FluoroView1000 microscope at a magnification of 60×. Mitochondrial membrane potential was determined by quantitation of MitoTracker Red fluorescence (Invitrogen), using the software ImageJ (Bethesda, MD) and the data were normalized by number of cells.

### Spectrophotometric analysis of CS activity

CS activity was measured in mitochondrial extracts obtained from fibroblasts (65,66), by determining 5,5"-dithiobis (2-nitrobenzoic) acid (DTNB) reduction at λ412 nm and calculated as nmol 2-nitro-5-thiobenzoate anion (TNB) min^−1^ · mg protein^−1^.

### FAO flux analysis

Flux through the FAO pathway was quantified by production of ^3^H_2_O from [9,10^−3^H] palmitate (PerkinElmer, Waltham, MA), conjugated to fatty acid-free albumin in fibroblasts cultured in a 24-well plate, as previously described (67). Palmitate bound to albumin was used at a final concentration of 12.4 μM (0.06 Ci/mmol). For each cell, FAO flux was measured in triplicate. The oxidation rates were expressed as pmol ^3^H-fatty acid oxidized/h/mg protein).

### ETF fluorescence reduction assay

The ETF reduction assay was performed using a Jasco FP-6300 spectrofluorometer (Easton, MD) with a cuvette holder heated with circulating water at 32°C. The assay was otherwise performed as described (68), at the indicated substrate concentrations. The enzyme was diluted 1200-fold into a buffer containing 50 mM Tris, pH 8.0, 5 mM EDTA and 50% glycerol, and 10 μL were used for each assay. The ETF concentration in the reaction mixture was 2 μM. Spectra Manager 2 software (Jasco, Inc.) was used to collect data and calculate reaction rate and Microsoft Excel was used to calculate the kinetic parameters.

### Cell viability assay

Cell viability was evaluated according to the instructions described using a 3-(4,5-dimethylthiazol-2-yl)-5-(3-carboxymethoxyphenyl)-2-(4-sulfophenyl)-2H-tetrazolium (MTS) assay kit (Abcam). The absorbance was read in a FLUOstar Omega plate reader at 490 nm.

### Apoptosis assay

Apoptosis was evaluated with an Alexa Fluor® 488 annexin V/Dead Cell Apoptosis kit according to manufacturer’s instructions (Invitrogen). The kit contains annexin V labeled with a fluorophore and propidium iodide (PI). Fluorescence was determined in a Becton Dickinson FACSAria II flow cytometer (BD Biosciences).

### Statistical analysis

Assays were performed in triplicate and the mean was used for statistical calculations. Statistical analysis was performed with GraphPad 5.0 software. Student’s *t*-test (independent) and Tukey multiple range test were applied for simple comparisons between groups. Differences were considered significant when *P <* 0.05.

## Supplementary Material

Supplementary Data
