# Supplementary material for: Mitochondrial energetics is impaired in very long-chain acyl-CoA dehydrogenase deficiency and can be rescued by treatment with mitochondria-targeted electron scavengers
Source: Hum Mol Genet. 2018 Nov 16;28(6):928–41. doi: 10.1093/hmg/ddy403 (PMC6400046; doi:10.1093/hmg/ddy403)
Supplement: Supplementary Data [file ddy403_supp.doc]

**Mitochondrial energetics is impaired in very long-chain acyl-CoA dehydrogenase deficiency and can be rescued by treatment with mitochondria-targeted electron scavengers**

Bianca Seminotti ^1,2^, Guilhian Leipnitz ^1,2^, Anuradha Karunanidhi ^1^, Catherine Kochersperger ^1^, Vera Y. Roginskaya ^3^, Shrabani Basu ^1^, Yudong Wang ^1^, Peter Wipf ^4^, Bennett Van Houten ^3^, Al-Walid Mohsen ^1,5^, Jerry Vockley ^1,5^*

^1^Division Medical Genetics, Department of Pediatrics, University of Pittsburgh, Pittsburgh, PA, 15224, USA; ^2^Programa de Pós-Graduação em Ciências Biológicas: Bioquímica, Departamento de Bioquímica, Instituto de Ciências Básicas da Saúde, Universidade Federal do Rio Grande do Sul, Porto Alegre, RS 90035-003, Brazil; ^3^Department of Pharmacology and Chemical Biology, University of Pittsburgh, Pittsburgh, PA 15213, USA; ^4^Department of Chemistry, University of Pittsburgh, Pittsburgh, PA 15260, USA; ^5^Department of Human Genetics, Graduate School of Public Health, University of Pittsburgh, Pittsburgh, PA 15213, USA

* To whom correspondence should be addressed at: Jerry Vockley, Division Medical Genetics, Department of Pediatrics, University of Pittsburgh, 4401 Penn Avenue, Pittsburgh, PA, 15224, USA. Telephone: 412-692-7746, Fax: 412-692-7816, E-mail: vockleyg@upmc.edu

**Supplementary Material**

Seminotti *et al.* **Mitochondrial energetics is impaired in very long-chain acyl-CoA dehydrogenase deficiency and can be rescued by treatment with mitochondria-targeted electron scavengers**

**Table S1.** Mutations of VLCAD deficient patients

| Patients | Genotype |
| --- | --- |
| FB671 | L540P  c.1707_1716dup |
| FB773 | c.848T>C V283A  c.1182+3G>T |
| FB833 | Heterozygote  c.520G>A (p.V174M)  c.1825G>A (p.E609K) |
| FB777 | Heterozygote  c.848T>C (p.V283A)  c.865G>A (p.G289R) |
| FB774 | G43D  L17F |
| FB780 | L202P  WT |

**Table S2.** Effect of *N*-acetylcysteine (NAC), Bezafibrate (Bez), Resveratrol (Resv), MitoQ and Trolox on superoxide levels in VLCAD deficient fibroblasts

| **Superoxide Production (MitoSOX Red, AFU)** | | | | |
| --- | --- | --- | --- | --- |
| Compound (time) | WT +  Vehicle | WT + Compound | VLCAD^1^ + Vehicle | VLCAD^1^ + Compound |
| NAC (48 h) | 3522 | 3192 | 9881^**^ | 10574^**^ |
| Bez (24 h) | 550 | 674 | 979^***^ | 2013^***^ |
| Resv (24 h) | 2064 | 2330 | 2807^**^ | 4252^**##^ |
| MitoQ (24 h) | 2443 | 13892 | 4287^***^ | 46198^****####^ |
| Trolox (48 h) | 2311 | 3452 | 4884^***^ | 8012^***###^ |

The cells were cultured in media without glucose for 48 h. Data are means ± SD. **P<0.01, ***P<0.001, ****P<0.0001, compared to WT; ^##^P<0.01, ^####^P<0.0001 compared to VLCAD deficient fibroblasts (Tukey multiple range test). ^1^ VLCAD deficient cell lines (FB671 and FB773).

**
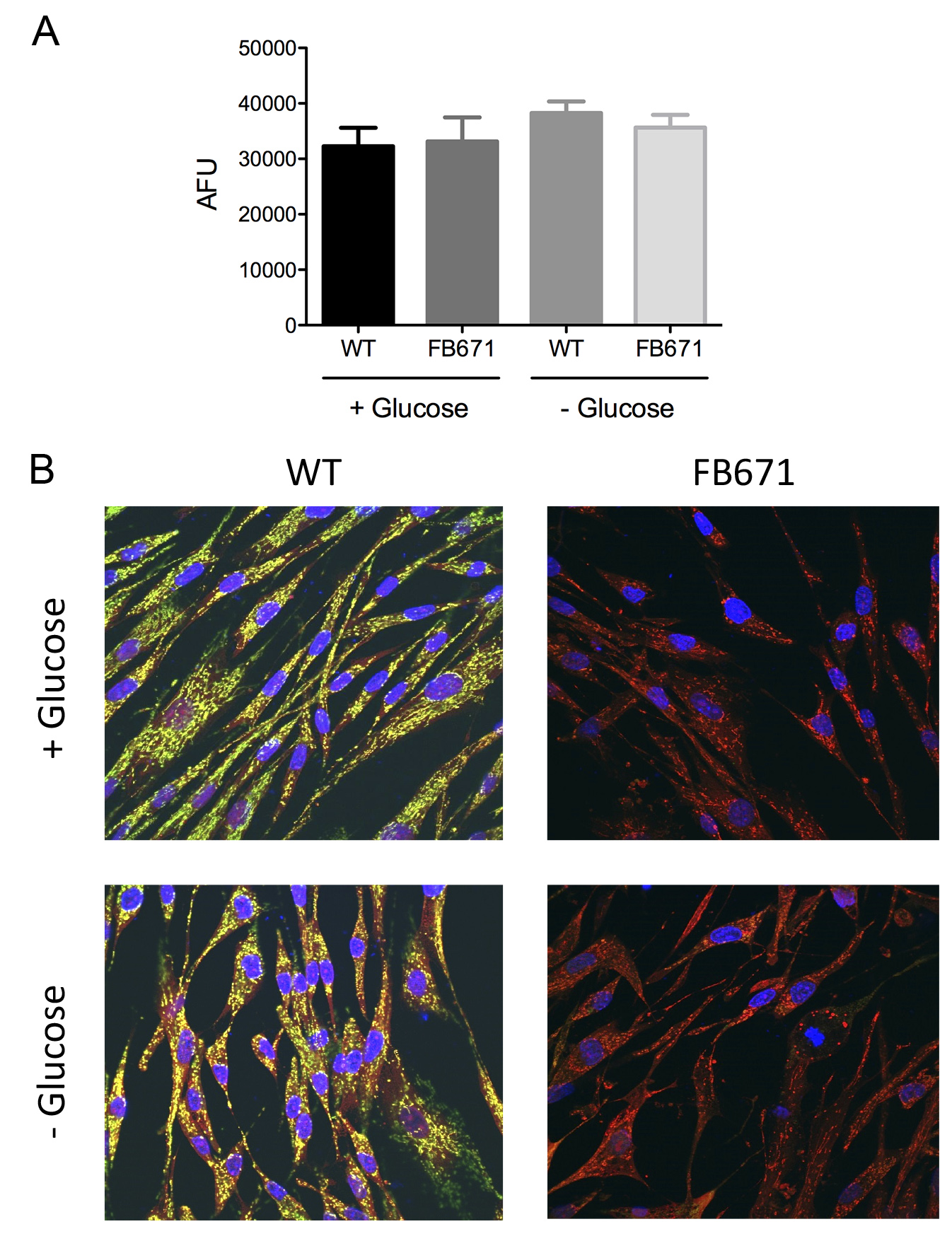
**

**Figure S1.** Mitochondrial membrane potential (ΔΨ) in FB671 fibroblasts cultured in media with or without glucose for 48 h. VLCAD deficient fibroblasts were incubated with MitoTracker Red. Data are means ± SD.

**
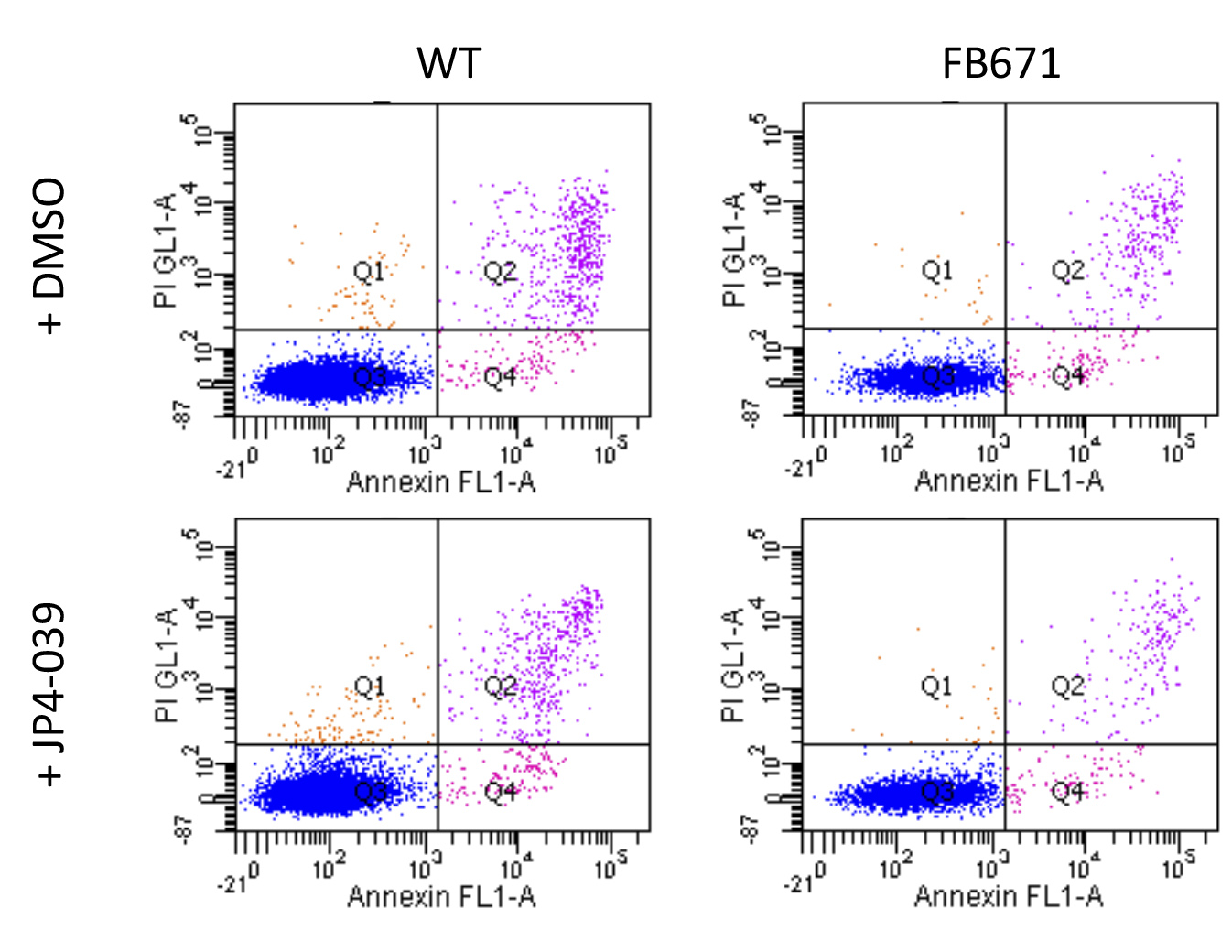
Figure S2.** Representative density plots of WT and FB671 fibroblasts (Annexin V versus Propidium iodide) measured by flow cytometry. VLCAD deficient cells were exposed to JP4-039 (40 nM) during 24 h before evaluating apoptosis.
